# Etiological spectrum, injury characteristics and treatment outcome of maxillofacial injuries in a Tanzanian teaching hospital

**DOI:** 10.1186/1752-2897-5-7

**Published:** 2011-06-02

**Authors:** Phillipo L Chalya, Mabula Mchembe, Joseph B Mabula, Emanuel S Kanumba, Japhet M Gilyoma

**Affiliations:** 1 Department of Surgery, Weill-Bugando University College of Health Sciences, Mwanza, Tanzania; 2 Department of Surgery, Muhimbili University of Health and Allied Sciences, Dar Es Salaam, Tanzania

**Keywords:** Maxillofacial injuries, etiology, injury characteristics, treatment outcome, Tanzania

## Abstract

**Background:**

Maxillofacial injuries pose a therapeutic challenges to trauma, maxillofacial and plastic surgeons practicing in developing countries. This study was carried out to determine the etiology, injury characteristics and management outcome of maxillofacial injuries at our teaching hospital.

**Patients and Methods:**

A prospective hospital based study of maxillofacial injury patients was carried out at Bugando Medical Centre from November 2008 to October 2009. Data was collected using a structured questionnaire and analyzed using SPPS computer software version 11.5.

**Results:**

A total of 154 patients were studied. Males outnumbered females by a ratio of 2.7:1. Their mean age was 28.32 ± 16.48 years and the modal age group was 21-30 years. Most injuries were caused by road traffic crushes (57.1%), followed by assault and falls in 16.2% and 14.3% respectively. Soft tissue injuries and mandibular fractures were the most common type of injuries. Head/neck (53.1%) and limb injuries (28.1%) were the most prevalent associated injuries. Surgical debridement (95.1%) was the most common surgical procedures. Closed reduction of maxillofacial fractures was employed in 81.5% of patients. Open reduction and internal fixation was performed in 6.8% of cases. Complications occurred in 24% of patients, mainly due to infection and malocclusion. The mean duration of hospital stay was 18.12 ± 12.24 days. Mortality rate was 11.7%.

**Conclusion:**

Road traffic crashes remain the major etiological factor of maxillofacial injuries in our setting. Measures on prevention of road traffic crashes should be strongly emphasized in order to reduce the occurrence of these injuries.

## Introduction

The maxillofacial region occupies the most prominent position in the human body and rendering it vulnerable to injuries quite commonly [1]. Maxillofacial injuries are commonly encountered in the practice of emergency medicine and are often associated with high morbidity resulting from increased costs of care and varying degrees of physical, functional and cosmetic disfigurement [2]. It is estimated that more than 50% of patients with these injuries have multiple trauma requiring coordinated management between emergency physicians and surgical specialists in otolaryngology, trauma surgery, plastic surgery, ophthalmology, and oral and maxillofacial surgery [3,4]. Maxillofacial injuries can occur as an isolated injury or may be associated with multiple injuries to the head, chest, abdominal, spinal and extremities [5].

The etiology of maxillofacial injuries varies from one country to another and even within the same country depending on the prevailing socioeconomic, cultural and environmental factors [2,6,7]. The relationship between alcohol consumption and maxillofacial injuries is well known [2,8,9].

The common etiologies of maxillofacial fractures, across the world, are road traffic accidents, falls, assaults, firearm injury, sports and industrial accidents [10-12]. Road traffic accident is reported to be the leading cause of maxillofacial fractures in developing countries [7,8,11,13], while interpersonal violence is the leading cause in developed countries [10]. The causes and pattern of maxillofacial injuries reflect trauma patterns within the community and, as such, can provide a guide to the design of programmes geared toward prevention and treatment [12].

Maxillofacial injuries involve soft and hard tissues injuries of face extending from frontal bone superiorly to mandible inferiorly and vary from soft tissue lacerations to complex fractures of maxillofacial skeleton [11]. The pattern of these injuries depends on the mechanism of mechanism of injury, magnitude and direction of impact force and anatomical site [2,7,11].

The management of injuries to the maxillofacial complex remains a challenge for oral and maxillofacial surgeons, demanding both skill and a high level of expertise [13,14]. Open reduction and internal fixation of maxillofacial fractures has been reported to results in a patient with a satisfactory facial appearance and restoration of function [14]. However, in resource-limited countries like ours, lack of expertise and facilities for open reduction and internal fixation and late presentation are a major problem in achieving acceptable cosmetic outcomes in maxillofacial trauma patients.

The vast majority of maxillofacial injuries are preventable; therefore, preventive strategies targeting at the etiology of these injuries is important in order to reduce their occurrence. A clear knowledge of injury characteristics and treatment outcome is vital in order to achieve acceptable functional and cosmetic outcomes.

The aim of this study was to describe our own experiences in the management of maxillofacial injuries outlining the etiological spectrum, injury characteristics and treatment outcome of these injuries in our local setting. The study provides basis for establishment of treatment guideline and planning for preventive strategies.

## Patients and Methods

In this prospective hospital based study, all consecutive maxillofacial injury patients admitted to the Accident & Emergency department of Bugando Medical Centre (BMC) over a one-year period from November 2008 to October 2009 were included. BMC is the only referral and teaching hospital in Mwanza, a city located in the north-western part of Tanzania along the shore of Lake Victoria. It is a teaching hospital for Weill-Bugando University College of Health Sciences and has the bed capacity of 1000.

Trauma patients are first seen at the A&E department where resuscitation is carried out according to Advanced Trauma Life Support (ATLS) principles. From the A&E department these patients are admitted in their respective surgical wards or ICU after definitive treatment.

During this study, all maxillofacial injury patients seen at the A&E department were, after informed written consent, consecutively recruited into the study. Patients who died before initial assessment and those without next of kin to consent were excluded from the study. Ethical approval to conduct the study was obtained from the WBUCHS/BMC joint institutional ethic review committee before the commencement of the study.

Information relevant to the study was obtained from the patient directly; when this was not possible, collateral history was obtained from either the police or relatives attending to the patients.

All maxillofacial bony injuries were diagnosed by conventional and panoramic radiographs. Advanced imaging techniques like computed tomography and magnetic resonance imaging were not used due to patients' financial constraints and their unavailability.

Data were collected using a pre-tested questionnaire. Data collected included: patient's demographic data, cause of injury, type of injury, time of injury, place of injury, status of prehospital care, mode of arrival in the hospital, associated injuries, severity of injury (GCS & ISS), treatment modalities and outcome of treatment (i.e. post-operative complications, length of hospital stay and mortality). The causes of injury were classified as road traffic accidents (RTAs), assault, falls, burn, sport related, animal bite and gunshot. The anatomic location of the mandibular fractures was classified according to Ivy and Curtis [15], while the maxillary fractures were classified as Lefort I, II, and III [16].

Data collected were analyzed using the statistical package for social sciences (SPSS) for Windows version 11.5. Data was summarized in form of proportions and frequency tables for categorical variables. Means, median and standard deviation were used to summarize continuous variables. A p-value of less than 0.05 was considered statistically significant.

## Results

During the period under study, a total of 154 patients were enrolled. 112 (72.7%) patients were males and females were 42 (27.3%) with a male to female ratio of 2.7:1. Their ages ranged from 6 to 71 years with a mean of 28.32 ± 16.48 years. The modal age group was 21-30 years. The majority of patients were unemployed (63.6%, n = 98) and most of them had either primary or no formal education (66.2%, n = 102).

The vast majority of injuries (77.9%, n = 120) were unintentional and the remaining 34 (22.1%) were intentional injuries mainly due to assault and interpersonal violence. There was no history of suicidal or indeterminate intent. The majority of patients 136 (88.3%) sustained blunt injuries and road traffic crash was the most common cause of injuries accounting for 88 (57.1%) of all injuries. Of these, 52 (59.1%) injuries were related to motorcycle accidents affecting motorcyclists, passengers and pedestrian. Table 1 shows distribution of patient according to the cause of injury. The majority of injuries occurred on roads, streets or highways (67.5%, n = 104) while 35 (22.7%), 8(5.2%) and 7(4.5%) injuries occurred at home, working place and recreational areas respectively. A history of alcohol use prior to injury was reported in 76 (49.4%) of cases.

**Table 1:** Distribution of patients according to cause of injury

Cause of injury	Frequency	Percentage
Road traffic Crash	88	57.1
Assault	25	16.2
Falls	22	14.3
Burn	12	7.8
Sport related	4	2.6
Animal bite	2	1.3
Gunshot injury	1	0.6

**Total**	**154**	**100**

The majority of patients (89.6%, n = 138) arrived to the Accident & Emergency department within 24 hours. Daytime injuries were recorded in 104 (67.5%) of patients while 50 (32.5% injuries occurred during the night.

None of our patients had pre-hospital care. The majority of them were brought in by relatives and Good Samaritan in 128 (83.1%). 26 (16.9%) patients were brought in by police. None of our patients were brought in by ambulance.

Of the 154 maxillofacial injuries, 142 (92.2%) were soft tissue injuries which included contusion, lacerations, abrasions and burn. The majority of soft tissue injuries (83.1%; n = 128) were located extra-orally. Maxillofacial fractures occurred in 54 (35.1%) patients, of these, the mandible was commonly involved in 38(70.4%) of patients (Table 2).

**Table 2:** Maxillofacial fractures (n = 54)

Fracture	Frequency	Percentage
**Mandibular fractures**	**38**	**70.4**
Angle	10	26.3
Condyle	8	21.1
Parasymphysis	8	21.1
Body	7	18.4
Symphysis	4	10.5
Ramus	1	2.6
Coronoid	-	-
**Nasal fractures**	**6**	**11.1**
**Maxillary fractures**	**4**	**7.4**
Le Fort I	1	25.0
Le Fort II	2	50.0
Le Fort III	1	25.0
**Zygomatic fractures**	**3**	**5.6**
**Orbital fractures**	**2**	**3.7**
**Frontal fractures**	**1**	**1.9**

Distribution of patients according to etiology and type of fracture is shown in Table 3.

**Table 3:** Distribution of patients according to etiology and type of fracture

Etiology	Type of fracture				
		
	Upper facial fractures	Mid-facial fractures	Lower-facial (mandibular) fractures	Panfacial fractures	Total
Road traffic crushes	1(1.9%)	4 (7.4%)	13(24.1%)	8(14.8%)	26
Assaults	-	2(3.7%)	9(16.7%)	2(3.7%)	13
Falls	-	2(3.7%)	8(14.8%)	1(1.9%)	11
Sports	-	1(1.9%)	3(5.6%)	-	4

**Total**	**1**	**9**	**33**	**11**	**54**

Sixty four patients (41.6%) had associated injuries. Of these, head/neck (53.1%) and musculoskeletal (28.1%) regions were commonly affected. (Table 4)

**Table 4:** Associated injuries (n = 64)

Associated injury	Frequency	Percentage
Head/neck injuries	34	53.1
Thoracic injuries	6	9.4
Abdominal injuries	5	7.8
Extremities injuries	18	28.1
Pelvic injuries	1	1.6

In patients who had associated head injuries, 34 patients (52.9%) had mild head injuries (Glasgow Coma Scale [GCS]: 13-15), 9 (26.5%) had moderate head injuries (GCS: 9-12), and 7 (20.6%) had severe head injuries (GCS: 3-8). Eleven missed maxillofacial injuries were recorded in eight (5.2%) patients who had associated head injuries.

Injury severity score (ISS) was calculated in 64 patients who had multiple injuries. The overall ISS ranged from 3-45 (mean 12.8). The majority of patients (62.5%; n = 40) had ISS < 16, whereas patients with ISS ≥16 were 24 (37.5%).

Surgical treatment was required in 103 (66.9%) of patients, with surgical debridement 98 (95.1%) being the most common surgical procedures performed. Closed method of fracture reduction was employed in 44(81.5%) of patients by mandibulo-maxillary inter-fixation, either with arch bars or eyelet wiring methods. Seven (6.8%) patients were managed by open reduction and internal fixation. Skin grafting of the facial burn wounds was performed in 5 (7.8%) patients.

Surgical treatment of the associated injuries included external fixators and open reduction and internal fixation (ORIF) for the limb fractures in 12 (18.8%) patients, craniotomy in 8 (12.5%) patients, exploratory laparotomy in 3 (4.7%) patients and tube thoracostomy in 2 (3.1%) patients.

The vast majority of patients (122; 79.2%) were admitted in general surgical wards and the remaining thirty-two (20.8%) patients were admitted in the intensive care unit (ICU) where 22 (68.8%) of them required ventilatory support. Twelve (7.8%) patients were later referred to other centers for more specialized care.

A total of 78 complications were recorded in 37 (24.0%). Of these, surgical site infection and malocclusion were the most prevalent complications in 24 (30.8%) and 18 (23.1%). Table 5

**Table 5:** Complications of maxillofacial injuries (n = 78)

Complications	Frequency	Percentage
Surgical site infection	24	30.8
Malocclusion	18	23.1
Keloids and hypertrophic scars	12	15.4
Chronic sinusitis	8	10.3
Permanent facial deformity	4	5.1
Non-union of mandibular fracture	4	5.1
Hemorrhage	3	3.8
Loss of smell sensation	3	3.8
Airway compromise	2	2.6

**Total**	**78**	**100**

The overall length of hospital stay ranged from 1 day to 70 days (mean = 18.12 ± 12.24 days). Patients with multiple maxillofacial fractures, associated injuries, maxillofacial burn and those with associated lower limb fractures had significantly longer hospital stay (*P *< 0.001).

In this study, eighteen patients died giving a mortality rate of 11.7%. Mortality rate was found to be significantly associated with age of the patient, presence of associated injuries, ISS ≥ 16, need for ventilatory support and presence of complications (*P *< 0.001).

## Discussion

The etiological factors and pattern of maxillofacial injuries have been reported to vary from one geographical area to another depending upon the socioeconomic status, geographic condition and cultural characteristics [2,6,7].

The male predominance in our study agrees with what is reported in literature [2,7-11,13,14,17]. Males are at greater risk due to their greater participation in high risk activities which increases their exposure to risk factors such as driving vehicles, sports that involve physical contact, an active social life and drug use, including alcohol.

In agreement with other studies [2,7-9,11,13,14,17], the majorities of patients in the present study were young adult in their third decade (21-30 years). However, this observation in contrast to some studies, where the dominant age groups having a high incidence were 0-10 years and 11-20 years respectively [17,18]. The possible reasons for the higher frequency of maxillofacial injuries in third decade may be attributed to the fact that people in this period of life are more active regarding sports, fights, violent activities, industry and high speed transportation. The low frequencies in the very young and old age groups are due to the low activities of these age groups.

The present study shows that the most common cause of maxillofacial injuries was road traffic accidents, which is consistent with other studies in developing countries [7,8,13,17,19], but in contrast to other studies done in developed countries which reported assaults as the most common cause of maxillofacial injuries [10,20-22]. These etiological differences reflect differences in socioeconomic factors, national infrastructure development (particularly roadways, traffic regulations and legislation), and other behavioral practices such as alcohol consumption and other criminal activities. The high number of maxillofacial injuries attributed to RTA in our study is attributed to recklessness and negligence of the driver, often driving under the influence of alcohol or drugs and complete disregard of traffic laws, over speeding, overloading, underage driving and poor conditions of roads and vehicles.

Excessive consumption of alcohol is strongly associated with facial injuries [10,23-25]. Alcohol impairs judgment, brings out aggression, often leads to inter-personal violence, and is also a major factor in motor vehicle accident [26]. In the present study, alcohol consumption prior to the injury was recorded in 49.4% of cases which is comparable to other studies [2,14]. Al Ahmed *et al *[13] in a review of 230 cases of maxillofacial injuries in Sharjah, United Arab Emirates reported no cases were associated with alcohol abuse. This discrepancy may be explained by differences between one country and another, in the strictness of laws governing the sale and consumption of alcohol which may be effective in preventing alcohol-related injuries.

The prehospital care of trauma patient has been reported to be the most important factor in determining the ultimate outcome after maxillofacial injuries [27]. None of our patients had pre-hospital care and the majority of them were brought in by relatives, Good Samaritan and police. None of these patients were brought in by ambulance. Similar observations had been reported in previous studies [5,7]. The lack of advanced pre-hospital care and ineffective ambulance system for transportation of patients to hospitals are a major challenges in providing care for trauma patients in our environment and have contributed significantly to poor outcome of these patients

Soft tissue injuries were the most frequently occurring type of injury and mandibular fracture was the most frequent type of bony injury. Similar finding was also reported by other studies [7,19,28]. This preponderance could be due to the fact that the mandible is the most prominent and only moveable facial bone, and hence has a greater chance of being fractured than the well-articulated mid-facial bones. Other studies reported midface fractures as the most frequent site of injury [11,29,30]. This difference in injury patterns reflects differences in the mechanism of injury and anatomical site of the fractured bone.

Head injury accounted for the greater majority of associated injuries and contributed significantly to missed maxillofacial injuries, similar to findings from other studies [31-33]. The incidence of missed injuries has been reported to be higher in patients with associated severe head injuries [31,33]. This is reflected in the high rate of missed maxillofacial injuries in our patients, the majority of them had associated severe head injuries. This finding calls for high index of suspicious when dealing with these patients.

There are many treatment regimens in maxillofacial fractures, but the treatment chosen may differ depending on many factors like cost of treatment, affordability by the patient, feasibility in the hospital, doctor's decision and skill, patient's willingness to avail the treatment advised - all of which may vary from one country to another. Majority of the patients treated in our hospital had closed reduction with arch bar fixation as the treatment and few patients were treated with open reduction and internal fixation, which is consistent with the studies conducted by Kamulegeya *et al *[7], Chandra [8], Erol *et al *[15] and Kilasara *et al *[27]. Open reduction and internal fixation has been reported to be the "gold standard" of treatment of maxillofacial fractures. However, this form of treatment has not become popular in our environment due to lack of expertise (i.e. maxillofacial surgeons) and facilities for open reduction and internal fixation are not readily available; and where available, the cost of treatment is usually quite prohibitive. Because of this shortcoming, the majority of patients with complex maxillofacial injuries are referred to the maxillofacial surgeons elsewhere for specialized care.

In various studies, complication rate ranges from 7 to 29% [34], and has been correlated to the severity of the fracture. In our study, the complication rate was found to be 24% which is higher than that found in by Kilasara *et al *[27] and lower than that found by Kamulegeya *et al *[7]. These differences in complication rates between these studies can be explained by differences in the severity of their fractures.

The high rates of infection in the present study could be ascribed to the use of closed reduction with mandibulo-maxillary inter-fixation (MMF) and its accompanying oral hygiene and nutritional challenges. Also, given the cost of dental care relative to the earning capacity of our patients, it was not surprising that many of them presented with very poor oral hygiene; we were not able to improve the situation before the MMF was performed.

The average length of hospital stay (LOS) in our study (18.12 days) was found to be longer than that of 2.5 days reported by Martins Junior et al [35]. The reason for this difference is that in the present study patients with multiple maxillofacial fractures, associated injuries, maxillofacial burn and those with associated lower limb fractures had significantly longer hospital stay contributing significantly to the overall mean LOS.

Our figure for mortality rate (11.7%) was found to be slight higher than that of 10.5% reported in Australia by Shahim *et al *[28]. The reason for high mortality rate in the present study is attributed to the following factors which were found to be statistically significantly associated with mortality: age of the patient, presence of associated injuries, Injury Severity Score ≥ 16, need for ventilatory support and presence of complications.

## Conclusion and Recommendation

Road traffic crush (RTC) was the major etiological factor of maxillofacial injuries in our setting and the young adult males were the main victims. Soft tissue injuries and mandibular fracture were the most frequently occurring type of injury. The majority of maxillofacial fractures were treated by closed method of fracture reduction. In the light of this study the following recommendations are given:

• To reduce the incidence of RTC, the laws regarding the precautions like seat belts, speed limits and traffic rules must be observed strictly

• An awareness campaign to educate the public especially the drivers about the importance of restraints and protective measures in motor vehicles should be started.

• Maxillofacial fractures should be managed by open reduction and internal fixation as early as possible in order to reduce the morbidity resulting from these injuries

## Competing interests

The authors declare they have no competing interest. The study had no external funding. Operational costs were met by authors

## Authors' contributions

PLC - Study design, data analysis, manuscript writing & editing and submission of manuscript, MM - Study design, data analysis, manuscript writing & editing, JBM - Data analysis, manuscript writing & editing. ESK - Data analysis & manuscript writing. SC - Data analysis, manuscript writing & editing. JMG - Study design, data analysis, manuscript writing & editing. All the authors read and approved the final manuscript.
